# Prevalence of Isolated Diastolic Hypertension and Associated Risk Factors among Different Ethnicity Groups in Xinjiang, China

**DOI:** 10.1371/journal.pone.0145325

**Published:** 2015-12-22

**Authors:** Fen Liu, Dilare Adi, Xiang Xie, Xiao-Mei Li, Zhen-Yan Fu, Chun-Fang Shan, Ying Huang, Bang-Dang Chen, Min-Tao Gai, Xiao-Ming Gao, Yi-Tong Ma, Yi-Ning Yang

**Affiliations:** 1 Xin Jiang Key Laboratory of Cardiovascular Disease, Clinical Medical Research Institute of First Affiliated Hospital of Xinjiang Medical University, Urumqi, China; 2 Department of Cardiology, First Affiliated Hospital of Xingjiang Medical University, Urumqi, China; 3 Baker IDI Heart and Diabetes Institute, Melbourne, Australia; Chang-Gung University, TAIWAN

## Abstract

**Objectives:**

Little is known about isolated diastolic hypertension (IDH) among different ethnicity groups. We aimed to investigate the prevalence and risk factors for IDH among the major ethnicity population i.e. Han, Uygur and Kazakh in Xinjiang, northwestern part of China.

**Methods:**

In total, 14,618 adult participants (7,799 males, 6,819 females) were recruited from the Cardiovascular Risk Survey conducted during 2007 and 2010. Blood pressure, body mass index and standard lipid profile and fasting glucose level from plasma were measured.

**Results:**

The overall prevalence of IDH was 10.8% in the Han, 4.5% in the Uygur and 8.7% in the Kazakh populations. When stratified by gender, IDH prevalence was 9.8% in men and 6.8% in women (*P<*0.001). The prevalence of IDH also varied significantly with age and it was highest in those aged 35–44 yrs old (9.7%) and lowest in those over 75 yrs old (4.1%, *P<*0.001). Multivariate logistic regression analysis showed that overweight (OR = 1.179, 95%CI: 1.015–1.369) or obesity (OR = 1.202, 95%CI: 1.015–1.424), smoking (OR = 1.362, 95%CI: 1.156–1.604) and high total cholesterol (TC) hyperlipidemia (OR = 1.237, 95%CI: 1.074–1.423) were significantly associated with a higher prevalence of IDH. Identified risk factors for IDH differed among ethnicity groups with male gender, young age (35–44 yrs old), more coffee or tea consumption and high TC hyperlipidemia in the Han; smoking and often coffee or tea consumption in the Uygur and male gender and overweight or obesity in the Kazakh populations.

**Conclusions:**

IDH prevalence in the Han population is higher than that in the Uygur and Kazak populations in Xinjiang, northwestern part of China. Male gender, middle age, overweight or obesity, smoking and high TC hyperlipidemia appear to be relevant risk factors of IDH in adults. Different ethnicity background had different sets of risk factors for IDH.

## Introduction

Epidemiological and clinical studies have demonstrated that hypertension is the leading modifiable risk factor for cerebro- and cardio-vascular diseases worldwide [1–2]. The prevalence of and mortality from hypertension are predicted to increase rapidly until at least 2025, and an estimated 560 million additional people will be affected by hypertension between 2000 and 2025 [3].

Isolated diastolic hypertension (IDH), an unrecognized form of hypertension, is defined as a diastolic blood pressure ≥90 mmHg while systolic blood pressure is <140 mmHg and is a consequence of an increase in arteriolar resistance [4–5]. IDH, although having a lower risk of cardiovascular mortality compared to systolic-diastolic hypertension (SDH) [6], is associated with an increase in cardiovascular risk, and patients with IDH are more likely to develop SDH or isolated systolic hypertension (ISH), which further increases the risk of stroke, coronary heart disease and end-stage renal disease [7].

IDH prevalence is higher among the younger population than systolic-diastolic hypertension (SDH), based on the Framingham Heart Study [8]. In addition, American National Health and Nutrition Examination Survey (NHANES III) showed that IDH is the most frequent form of hypertension in the population <40 yrs old and is comparable to SDH in its prevalence in individuals aged between 40–49 yrs old [9,10]. The accumulated evidence from a number of large population studies, including the Framingham study, suggests that lack of exercise, larger BMI, higher glucose and uric acid concentrations, smoking and drinking could be potential risk factors for IDH incidence [11–12]. Subjects with IDH, although constituting a large portion of the hypertensive population (14–24%), have been shown to be less likely to receive anti-hypertensive treatment than those with ISH or SDH [6, 13]. Furthermore, no robust epidemiological data are available to estimate IDH prevalence in population with different ethnic backgrounds. Hence, the purpose of the current study was to investigate IDH prevalence and to explore its associated risk factors in different ethnicities, i.e. Han, Uygur and Kazakh in Xinjiang, northwestern part of China.

## Methods

### Ethics statement

This study was approved by the ethics committee of the First Affiliated Hospital of Xinjiang Medical University and was conducted according to the standards of the Declaration of Helsinki. Written informed consent was obtained from each participant to collect the relevant clinical data.

### Participants

All of the participants were selected from the Cardiovascular Risk Survey (CRS) conducted during October 2007 and March 2010. A detailed description of study population and methods were previously reported [14–15]. Briefly, the CRS consisted of 16,460 adults aged ≥35 years old, of whom 14,618 subjects (5,757 Han, 4,767 Uygur and 4,094 Kazakh Chinese) completed the survey, yielding a response rate of 88.8%. The CRS was a multiple ethnicity, community-based and cross-sectional study. It was designed to investigate the prevalence and risk factors for cardiovascular disease and to determine genetic and environmental contributions to atherosclerosis, coronary artery disease and stroke in three major ethnicity groups, i.e. the Han, Uygur, and Kazakh populations in Xinjiang from 6 different administrative regions including Urumqi, Kelamayi, Fukang, Turpan, Hetian and Yili prefectures. Individuals with multi-organ failure syndrome and incomplete data were excluded from this survey.

### Data collection and Biochemical analysis

A standard questionnaire was used to collect general personal information and medical history as previously described [16]. Height and body weight were measured using standard methods. Smoking and drinking conditions were self-reported. After 12 h of overnight fasting, 5 mL of venous blood was collected into tubes containing ethylene diamine tetra acetic acid and processed to obtain plasma within 4 h in the examination centers of local hospitals in the participant’s residential area. Standard biochemical analysis using AR/AVL Clinical Chemistry System (Dimension, Newark, NJ, USA) were performed in the Clinical Laboratory Department of the First Affiliated Hospital of Xinjiang Medical University. Biochemical markers in plasma including total cholesterol (TC), triglycerides (TG), fasting blood glucose (FBG), high-density lipoprotein cholesterol (HDL-c), low-density lipoprotein cholesterol (LDL-c), blood urea nitrogen (BUN), creatinine (Cr) and uric acid (UA) were measured.

### Blood pressure measurements

A mercury sphygmomanometer was used to measure blood pressure while the patient was in a sitting position after a 15 min resting period during a 30 min-period. Subjects were required to refrain from smoking or consuming caffeine. The appearance of the first sound was used to define systolic blood pressure (SBP), and the disappearance of sound was used to define diastolic blood pressure (DBP). Two measurements were performed with an interval of 15 min Readings for SBP and DBP were recorded and averaged for data analysis. If the first two measurements in either SBP or DBP differed by more than 5 mmHg, additional measurement was taken.

### Definition of hypertension

Hypertension was defined in accordance with the 2010 guidelines for hypertension prevention and control [17] when SBP was ≥140 mmHg and/or DBP was ≥90 mmHg without taking anti-hypertensive medications or there was a history of hypertension with daily or regular (≥3 days per week) use of anti-hypertensive medications within a year. The subtypes of hypertension were further defined as IDH with SBP<140 mmHg and, DBP ≥90 mmHg; ISH with SBP ≥140 mmHg and DBP <90 mmHg; and SDH with SBP ≥140 mmHg and DBP ≥90 mmHg in two measurements or when there was antihypertensive treatment.

### Definition of risk factors

Dyslipidemia was defined as having one of the following four lipid abnormalities or according to self-report of using anti-hyperlipidemic medications. TC concentration >6.22 mmol/L (240 mg/dl) was defined as hypercholesterolemia; TG >2.26 mmol/L (200 mg/dl) was defined as hypertriglyceridemia; LDL-c concentration >4.14 mmol/L (160 mg/dl) was defined as high LDL-c; and HDL-c concentration <1.04 mmol/L (40 mg/dl) was defined as low HDL-c [18].

Diabetes mellitus was defined as the presence of active treatment with insulin or oral anti-diabetic agents or by positive self-reported response for current diabetes treatments in the survey. For patients on dietary treatments, documentation of an abnormal FBG ≥7.0 mmol/L (≥126 mg/dL) or glucose tolerance test based on WHO criteria [19] was required to establish this diagnosis.

Body mass index (BMI) was calculated by dividing the body weight in kilograms by the height in meters squared. BMI ≥25–29.9 kg/m^2^ was defined as overweight and BMI ≥30 kg/m^2^ was defined as obesity [20].

Participants reporting regular smoking in the previous 6 months were considered as current smokers and who were regular drinking in the last 6 months were considered as alcohol users. For measuring coffee or tea consumption, which was divided into 3 categories of never, occasionally (1–4 cups/day) and often (≥5 cups/day) [21].

### Statistical analysis

The data were verified and corrected by 2 staff members using EpiData3.02 software (EpiData Association, Odense, Denmark). The statistical analysis was conducted using Social Sciences-SPSS for Windows version 17.0 (SPSS, Inc., Chicago, IL, USA). Continuous variables were expressed as mean±standard deviation (SD), numerical data were expressed as rates, and a chi-square test (*χ* ^*2*^) was used to evaluate differences between groups. Age standardization was performed according to the Census of Xinjiang Uygur autonomous region in 2010. The risk factors for IDH were analyzed using a multivariate unconditional logistic regression, and the significance level alpha value was set to 0.05.

## Results

### General characteristics of study participants

General characteristics of study participants are presented in Table 1. A total of 14,618 participants, comprised of 6,819 (46.6%) men and 7,799 (53.4%) women, were recruited into the study. Among these participants, 5,757 (39.4%) were Han, 4,767 (32.6%) were Uygur and 4,094 (28.0%) were Kazakh ethnicities. There were differences in age, BMI, SBP, DBP, FBG, drinking, smoking, coffee/tea consumption and plasma levels of TG, TC, HDL-c, BUN, Cr and UA among the 3 ethnicities, while there was no significant difference in the level of LDL-c among three ethnicities. The average age of total study participants was 50.6±12.5 yrs with Kazakh participants being younger than Han and Uygur participants (*P<0*.*05*). BMI was the lowest in Han but the largest in Kazakh participants. For SBP and DBP, Kazakh participants had the highest BP which were significantly higher than that in Han and Uygur participants (both *P<0*.*05*). Smoking and drinking had the highest proportion in Han, and the proportion of often coffee or tea consumption was highest in the Kazakh population. The plasma levels of TC and HDL were the highest in the Kazakh, while TG was lowest in the Kazakh among the three ethnicities. For kidney function, the BUN and Cr were lowest in the Kazakh and highest in the Uygur and Han participants, respectively. Finally, UA level was the highest in the Han and the lowest in the Uygur participants.

**Table 1:** General characteristics of study participants.

Group	Han (n = 5757)	Uygur (n = 4767)	Kazakh (n = 4094)
Age, years	52.5±12.7	50.7±13.0*	48.6±11.7* †
BMI, kg/m^2^	25.13±3.50	25.84±4.42*	26.56±4.76* †
SBP, mmHg	133±20	131±21*	140±25* †
DBP, mmHg	85±16	80±15*	88±20* †
FBG, mmol/l	5.34±1.78	4.94±1.66*	5.13±1.51* †
Drinking	1098 (19.1%)	466 (9.8%)*	587 (14.3%)* †
Smoking	1767 (30.7%)	845 (17.7%)*	203 (5.0%)* †
Coffee/tea consumption			
Never	2245 (39.0%)	845 (17.7%)*	203 (5.0%)* †
Occasionally	1783 (31.0%)	1119 (23.5%)*	120(2.9%)* †
Often	1729 (30.0%)	2803 (58.8%)*	3771 (92.1%)* †
TG, mmol/l	1.72±1.45	1.64±1.22*	1.21±0.93*/†
TC, mmol/l	4.69±1.08	4.36±1.13*	4.78±1.16* †
HDL-c, mmol/l	1.26±0.45	1.26±0.47	1.29±0.43* †
LDL-c, mmol/l	2.87±0.91	2.87±0.92	2.90±0.93
BUN, mmol/L	4.94±1.47	5.24±1.71*	4.66±1.53* †
Cr, umol/L	75.83±26.14	71.42±29.68*	70.48±19.80*
UA, umol/L	306.24±86.85	249.44±76.11*	259.82±78.74* †

BMI, body mass index; SBP, systolic blood pressure; DBP, diastolic blood pressure; FBG, fasting blood glucose; TG, triglycerides; TC, total cholesterol; HDL-c, high density lipoprotein-cholesterol; LDL-c, low density lipoprotein-cholesterol; BUN, blood urea nitrogen; Cr, creatinine; UA, uric acid

*P<0.*05* vs. the Han participants

† *P<0*.*05* vs. the Uygur participants.

Further, the general characteristics of IDH positive and negative participants were also presented in S1 Table. There were significant differences in age, SBP, DBP, drinking, smoking, TG, TC and UA, while there was no difference in BMI, FBG, HDL-c, LDL-c, BUN, and Cr between the two subgroups. Age and SBP were lower in IDH positive compared to IDH negative group, the proportion in drinking and smoking were higher in IDH positive group. Plasma levels of TG, TC and UA were also higher in IDH positive group (all *P<0*.*05*).

### Prevalence of IDH

**Fig 1:**
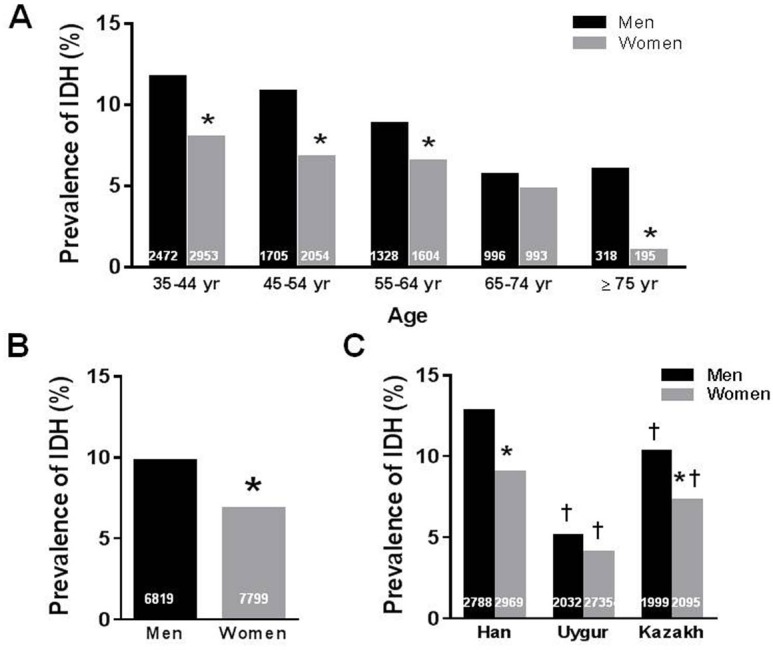
The prevalence of isolated diastolic hypertension (IDH) in men and women with different ages and ethnicities. **A**. Gender difference of IDH in different age groups. *P<0.*001* vs. men in the same group. **B**. Overall gender difference in IDH. *P<0.*001* vs. men. **C.** Gender difference of IDH in different ethnicities. The number at the bottom of each bar indicates the group size. *P<0.*001* vs. men in the same group. †*P<0*.*001* vs. Han or other groups in the same gender.

IDH was defined in 1197 participants (men n = 666, women n = 531) from the total study population, and the overall prevalence of IDH was 8.2%. IDH prevalence in the different ethnicities was found to be the highest in Han (10.8%) and the lowest in Uygur participants (4.5%). It was 8.7% in Kazakh participants, and there was a statistical significance among the three ethnic groups (*P<0*.*001*). Further, the prevalence of IDH in different age groups also varied significantly (*P<0*.*001*), with the highest prevalence in participants aged 35–44 yrs old and the lowest prevalence in men aged ≥65 yrs old (5.7%) and especially in women aged ≥75 yrs old (1.0%, Fig 1A). When stratified by gender, IDH prevalence was 9.8% in men and 6.8% in women, showing a significant difference between genders (*P<0*.*001*. Fig 1B). Further, the IDH prevalence for men was 11.7%, 10.8%, 8.8%, 5.7% and 6.0% in 35–44 yrs, 45–54 yrs, 55–64 yrs, 65–74 yrs and ≥75 yrs age groups, respectively (*P<0*.*001*), and it for women was 8.0%, 6.8%, 6.5% 4.8% and 1.0% for the same corresponding age groups (*P<0*.*001*. Fig 1A), indicating a higher IDH prevalence in men than in women cross different age groups. While a 12.8% and 10.3% IDH prevalence in male Han and Kazakh versus 9.0% and 7.3% in female Han and Kazakh participants were observed, there was no gender difference in Uygur participants (Fig 1C).

### Identification of IDH risk factors

To identify potential risk factors of IDH, we compared multiple factors listed in Table 1 between IDH positive (n = 1197) and negative participants (n = 13421) using multivariate unconditional logistic regression analysis, statistical results are presented in Table 2. Interestingly, we found that young adults had a higher risk to develop IDH. Using ≥75 yrs age group as a reference [odds ratio (OR) = 1], we found that compared with other age groups, the ratio of IDH positive participants was significantly higher in 35–44 yrs age group (OR = 2.753, 95% CI: 1.735–4.370), whereas in 65–74 yrs age group, the risk decreased by 1.28-fold (OR = 1.279, 95% CI: 0.780–2.098). Further, the risk of IDH elevated with increasing BMI, and it was 1.18-fold higher in overweight participants (OR = 1.179, 95% CI: 1.015–1.369) and 1.20-fold higher in obesity participants (OR = 1.202, 95% CI: 1.015–1.424). In addition, smoking (OR = 1.362, 95% CI: 1.156–1.604) and high TC hyperlipidemia (OR = 1.237, 95% CI: 1.074–1.423) were also associated with a higher risk of IDH.

**Table 2:** Multivariate unconditional logistic regression analysis risk factors of isolated diastolic hypertension in study participants.

Factor	B	S.E.	Wald	df	P	OR	95% CI
Sex	-0.162	.081	4.033	1	*0*.*045*	-0.850	0.726–0.996
Age			57.606	4	*0*.*000*		
More than 75y						1	
35–44 y	1.013	0.236	18.460	1	*0*.*000*	2.753	1.735–4.370
45–54 y	0.880	0.238	13.667	1	*0*.*000*	2.412	1.512–3.847
55–64 y	0.726	0.241	9.042	1	*0*.*003*	2.067	1.288–3.318
65–74 y	0.246	0.252	0.953	1	*0*.*329*	1.279	0.780–2.098
Ethnic			97.239	2	*0*.*000*		
Han						1	
Uygur	0.303	0.090	11.479	1	*0*.*000*	0.001	1.136–1.614
Kazakh	-0.571	0.099	33.468	1	*0*.*000*	0.565	0.466–0.685
Smoking	0.039	0.084	13.666	1	*0*.*000*	1.362	1.156–1.604
Drinking	0.024	0.092	0.067	1	*0*.*796*	1.024	0.855–1.226
Coffee/Tea consumption			1.095	2	*0*.*578*		
Never						1	
Occasionally	0.056	0.095	0.346	1	*0*.*556*	1.057	0.878–1.273
Often	-0.037	0.089	0.176	1	*0*.*675*	0.963	0.808–1.148
BMI			6.062	2	*0*.*048*		
Normal						1	
Overweight	0.165	0.076	4.663	1	*0*.*031*	1.179	1.015–1.369
Obesity	0.184	0.086	4.557	1	*0*.*033*	1.202	1.015–1.424
Diabetes	-0.138	0.140	0.982	1	*0*.*322*	0.871	0.662–1.145
TG	0.044	0.075	0.000	1	*0*.*988*	0.999	0.862–1.157
TC	0.212	0.072	8.775	1	*0*.*003*	1.237	1.074–1.423
HDL-c	0.044	0.069	0.406	1	*0*.*524*	1.045	0.912–1.197
LDL-c	-0.047	0.067	0.478	1	*0*.*489*	0.954	0.836–1.089
Constant	-3.379	0.262	165.279	1	*0*.*000*	0.034	

BMI, body mass index; TG, triglycerides; TC, total cholesterol; HDL-c, high density lipoprotein-cholesterol; LDL-c, low density lipoprotein-cholesterol.

### Comparison of identified IDH risk factors in the three ethnicity groups

After the finding of the difference in IDH prevalence among the three ethnicities, we applied multivariate unconditional logistic regression analysis on all identified risk factors and attempted to identify any existing difference in these risk factors which may explain the difference in IDH prevalence among these ethnicities. The comparison was made between IDH positive participants in Han (n = 622), Uygur (n = 215) and Kazakh (n = 356) and their corresponding IDH negative participants and the results are shown in Table 3. In the Han population, gender (male, OR = 0.782, 95% CI: 0.615–0.995), middle age (35–44 yr group, OR = 4.160, 95% CI: 2.169–7.980), often coffee or tea consumption (OR = 0.794, 95% CI: 0.636–0.991) and TC hyperlipidemia (OR = 1.436, 95% CI: 1.185–1.740) remained as risk factors; In the Uygur population, smoking (OR = 3.626, 95% CI: 2.295–5.729) and more coffee or tea consumption (OR = 2.876, 95% CI:1.671–4.950) remained as risk factors and in the Kazakh population, gender (male, OR = 0.691, 95% CI: 0.529–0.901) and overweight or obesity (OR = 1.719, 95% CI: 1.278–2.311) remained as risk factors for IDH.

**Table 3:** Multivariate unconditional logistic regression analysis for risk factors of isolated diastolic hypertension in the three ethnicities.

	Han			Uygur			Kazakh		
	OR	95% CI	P	OR	95% CI	P	OR	95% CI	P
Sex	0.782	0.615–0.995	*0*.*045*	1.712	1.139–2.575	*1*.*712*	0.691	0.529–0.901	*0*.*006*
Age			*0*.*000*			*0*.*002*			*0*.*194*
More than 75y	1			1			1		
35–44 y	4.160	2.169–7.980	*0*.*000*	1.579	0.623–3.998	*0*.*335*	1.742	0.686–4.427	*0*.*243*
45–54 y	2.981	1.540–5.771	*0*.*001*	1.870	0.736–4.751	*0*.*188*	1.703	0.665–4.358	*0*.*267*
55–64 y	2.818	1.446–5.490	*0*.*002*	1.054	0.404–2.748	*0*.*914*	1.593	0.615–4.125	*0*.*338*
65–74 y	1.723	0.870–3.414	*0*.*119*	0.595	0.202–1.753	*0*.*347*	1.028	0.375–2.823	*0*.*957*
Smoking	1.242	0.970–1.591	*0*.*085*	3.626	2.295–5.729	*0*.*000*	1.105	0.850–1.436	*0*.*456*
Drinking	1.051	0.825–1.339	*0*.*689*	1.017	0.635–1.628	*0*.*944*	0.816	0.579–1.151	*0*.*816*
Coffee/Tea consumption			*0*.*124*			*0*.*001*			*0*.*162*
Never	1			1			1		
Occasionally	0.916	0.741–1.132	*0*.*416*	2.728	1.517–4.905	*0*.*001*	1.218	0.579–2.564	*0*.*603*
Often	0.794	0.636-.991	*0*.*041*	2.876	1.671–4.950	*0*.*000*	0.755	0.460–1.240	*0*.*267*
BMI			*0*.*187*			*0*.*665*			*0*.*002*
Normal	1			1			1		
Overweight	0.989	0.805–1.215	*0*.*914*	1.098	0.779–1.549	*0*.*593*	1.719	1.278–2.311	*0*.*000*
Obesity	1.217	0.949–1.562	*0*.*122*	0.936	0.638–1.372	*0*.*733*	1.485	1.089–2.027	*0*.*013*
Diabetes	.834	0.588–1.183	*0*.*309*	1.154	0.636–2.094	*0*.*636*	0.822	0.422–1.600	*0*.*564*
TG	1.065	0.875–1.296	*0*.*529*	1.049	0.762–1.446	*0*.*769*	0.843	0.605–1.175	*0*.*314*
TC	1.436	1.185–1.740	*0*.*000*	1.131	0.784–1.631	*0*.*510*	0.918	0.713–1.182	*0*.*507*
HDL-c	0.980	0.809–1.186	*0*.*834*	1.208	0.889–1.641	*0*.*228*	1.105	0.859–1.422	*0*.*437*
LDL-c	0.991	0.823–1.192	*0*.*921*	1.095	0.811–1.479	*0*.*555*	0.809	0.633–1.034	*0*.*091*

BMI, body mass index; TG, triglycerides; TC, total cholesterol; HDL-c, high density lipoprotein-cholesterol; LDL-c, low density lipoprotein-cholesterol.

## Discussion

Hypertension is the leading risk factor for cerebro- and cardio-vascular mortality and disability worldwide, and it kills approximately 9.4 million people annually [22]. The prevention, treatment, and control of hypertension have already become major public health challenges [23], whereas the added risks that are conferred by subtypes of hypertension, especially IDH, have not been well described, and very few epidemical data are available on IDH prevalence in different ethnicity groups, including those in Xinjiang, northwest part of China. The results from our study indicate that IDH prevalence among adult populations in Xinjiang, which is located in the center of Asia, varies in different ethnic groups. Overall, 10.8% of the Han population, 4.5% of the Uygur population and 8.7% of the Kazakh population have IDH, and differences in ethnicity background were associated with different sets of risk factors for IDH, according to the data in our study.

Several previous large and cross-sectional studies have reported on IDH prevalence in China and other countries and have indicated that that prevalence of IDH fluctuates between 3.6% and 6.2% in the general population, and is as high as 23.7% in patients with untreated metabolic syndrome [14, 24–28]. Abiodun et al [24] reported that subjects with IDH accounted for 4.8% of the total population in Ibadan. Midha et al [5] showed that prevalence of IHD in the adult population of the Kanpur district was 4.5% with 6.2% in men and 3.1% in women. In China, a number of studies have analyzed the prevalence of IDH and its risk factors. For example, Huang et al first reported that the prevalence of IDH was 4.4% in China in 2004 [26]. Kelly and Gu et al conducted a prospective cohort study including 169,871 Chinese men and women aged ≥40 yrs, and they found that the overall prevalence of IDH was 3.6% [14]. Wu et al showed that the incidence of IDH in Tongshan County of Jiangsu Province, China, was 6.24±0.17% [27]. In addition, Yeh et al reported, in the Cardiovascular Disease Risk Factors Two-Township Study, that the peak prevalence of IDH occurred in adults aged 35–49 yrs old was 14.5 per 1,000 person-year for men and 4.2 per 1,000 person-year for women [28]. The prevalence of IDH, however, in the minority populations of China has been rarely reported. In our study, we used CRS data to investigate the prevalence of IDH in Xinjiang, a remote and multi-ethnicity region containing a total of 47 ethnicities, 13 of which are minorities. The Han, Uygur and Kazakh populations are the three major ethnic groups in this region. The Han Chinese accounted for 40% of the total population, whereas minorities, especially the Uygur and Kazakh Chinese, collectively accounted for another 40% of the total population. These ethnic groups have unique lifestyles and nature environment which are different from the Chinese in other parts of China. Our results indicate that the prevalence of IDH in Uygur and Kazakh populations is significantly lower than that in Han population in Xinjiang. Interestingly, the prevalence of IDH in the Han population estimated in the present study exceeds the average level in the general Chinese population that has been reported by others.

Among subtypes of hypertension, IDH is more prevalent in younger adults, with an average age of 40–45 years old for both genders and more prevalent in males. Biologically, in this age group, large artery elasticity functions well and only peripheral vascular resistance can increase diastolic blood pressure [29]. Consistent with these findings, our study showed that the prevalence of IDH is higher in the 35–44 yrs age group. Notably, IDH was more common in men (9.8%) than in women (6.8%) in our study, which is consistent with other reports [5, 8, 25–26]. We also observed a significantly lower prevalence of IDH in women than in men in the ≥75 yrs age group, that is different from another study on Han Chinese living in southeastern part of China with comparable prevalence of IDH between both genders in the similar age [26]. In that study they did not provide group size and there was few study reported IDH prevalence in such old age group. In regard to the relative small sample size in this age group in our current study, we could not pinpoint the responsible factors for such discrepancy.

Our data also illustrated that increasing BMI, smoking and high TC hyperlipidemia were associated with a higher risk of IDH. Obesity is an increasing epidemic in both adults and children, and it is common to see obese individuals with concomitant hypertension. Increased blood viscosity is associated with obesity and may, by increasing the rheological component of peripheral resistance, contribute to obesity-associated changes in arterial blood pressure [30]. In our study, both overweight and obesity were associated with a higher risk of IDH, which is similar as the results of the Framingham study [31]. A growing body of research on the effects of smoking on arterial compliance supports the association between smoking and IDH, suggesting that smoking elevates DBP by increasing arterial stiffness even in younger adults [32–33]. Although there have been inconsistencies in this finding among previous studies [31, 34–35]. Multivariate analysis in our study indicated that IDH was positively associated with smoking. Dyslipidemia often coexists with hypertension, and in our study, a high TC was also a risk factor for IDH, but other lipid profiles were not. Contrary to the findings of previous studies [35], we did not observe an association between incidence of IDH and high levels of blood glucose. Importantly, we observed that the risk factors for IDH were different among the three ethnicities. In the Han participants, middle age, more coffee or tea consumption and high TC hyperlipidemia were risk factors; in Uygur participants, smoking and often coffee or tea consumption were risk factors and in the Kazakh participants, overweight or obesity were risk factors. Compared to Uygur and Kazakh IDH positive participants, the Han counterparts seem to have more potential risk factors, which was coupled with a higher IDH prevalence. This information may indicate a more susceptibility of Han population to IDH. These differences might attribute to socioeconomic conditions, lifestyle (e.g., eating habits) and genetic factors. A further study is required to better define the risk factors responsible for a higher prevalence of IDH.

It is worthwhile to mention that the epidemiological profile of IDH in the current study is different from what we observed in ISH previously. In our previous study [36] we found that the prevalence of ISH was 12.4% in the Han, 11.75% in the Uygur and 11.50% in the Kazakh populations, which were higher than the prevalence of IDH in all three ethnicity groups. Moreover, ISH was more common in women than in men in Han participants, but there were no significant gender difference in the Uygur and Kazakh populations. This is different from IDH with more susceptibility in men in all the three ethnicities. Further, there are some overlapping in regard to potential risk factors for both ISH and IDH. We observed previously that overweight, obesity, diabetes, high TG and TC hyperlipidemia were associated with higher risk of ISH. Here we found that increasing BMI, male gender, middle age, more coffee or tea consumption, smoking and high TC hyperlipidemia were associated with higher risk of IDH. These results imply that overweight and high TC hyperlipidemia are the common risk factors for both ISH and IDH.

This is the first cross-sectional study to provide insight on the prevalence of IDH and potential risk factors in the three major different ethnicities in Xinjiang, China. However, there are some limitations existing in the current study. First, our study is retrospective and cross-sectional, such nature of the study precludes the possibility of determining any causal association between IDH and related risk factors. Second, we failed to analyze information related to dietary patterns (e.g., salt consumption and eating habits), physical activity and socioeconomic conditions (including lower household income, lower educational attainment, and lack of health insurance) in the study population. Third, data on alcohol consumption, smoking, or the amount and type of tea or coffee consumed were based on self-reporting and therefore there is a possibility of misclassification of exposure. A further prospective study in a larger scale of population will help to better elucidate the epidemiological picture of IDH and related risk factors.

In conclusion, there is an ethnicity difference in the prevalence of IDH in Xinjiang, northwestern part of China. Among the three studied populations, the Han Chinese had the highest IDH prevalence and it exceeds the average level in other Chinese populations reported previously. While the Uygur Chinese had the lowest IDH prevalence. Male gender, middle age, overweight or obesity, smoking and high TC hyperlipidemia appear to be relevant risk factors of IDH in adults. Different ethnicity background had different sets of risk factors for IDH.

## Supporting Information

S1 Table (DOCX)
