# Supplementary material for: Prevalence of Isolated Diastolic Hypertension and Associated Risk Factors among Different Ethnicity Groups in Xinjiang, China
Source: PLoS One. 2015 Dec 22;10(12):e0145325. doi: 10.1371/journal.pone.0145325 (PMC4690591; doi:10.1371/journal.pone.0145325)
Supplement: S1 Table — (DOCX) [file pone.0145325.s001.docx]

**Supporting Information**

**S1 Table.** General characteristics of isolated diastolic hypertension (IDH)

positive and negative participants.

|  | IDH negative | IDH positive |
| --- | --- | --- |
| N= | 13421 | 1197 |
| Age, years | 51.0±12.8 | 49.2±10.4* |
| BMI, kg/m2 | 25.8±4.3 | 25.8±3.7 |
| SBP, mmHg | 134.9±23.1 | 129.6±6.8* |
| DBP, mmHg | 83.1±17.1 | 97.7±6.2* |
| FBG, mmol/l | 5.1±1.7 | 5.2±1.6 |
| Drinking | 1891(14.1) | 260(21.7) * |
| Smoking | 3718(27.8) | 451(37.7) * |
| Coffee/Tea consumption |  |  |
| never | 3011(22.4%) | 282(23.6%) |
| occasionally | 2763(20.6%) | 259(21.6%) |
| often | 7647(57.0%) | 656(54.8%) |
| TG, mmol/l | 1.5±1.3 | 1.6±1.3* |
| TC, mmol/l | 4.6±1.1 | 4.8±1.0* |
| HDL-c, mmol/l | 1.3±0.5 | 1.3±0.4 |
| LDL-c, mmol/l | 2.9±0.9 | 2.8±0.9 |
| BUN, mmol/L | 4.96±1.59 | 4.92±1.50 |
| Cr, umol/L | 72.86±26.44 | 73.46±19.47 |
| UA, umol/L | 273.70±85.16 | 286.71±84.85* |

BMI, body mass index; SBP, systolic blood pressure; DBP, diastolic blood pressure; FBG, fasting blood glucose; TG, [triglyceride](app:ds:triglyceride)s; TC, total cholesterol; HDL-c, high density lipoprotein-cholesterol; LDL-c, low density lipoprotein-cholesterol; BUN, blood urea nitrogen; Cr, creatinine; UA, uric acid; **P<0.05* vs. the negative participants.
